# Rotenone Upregulates Alpha-Synuclein and Myocyte Enhancer Factor 2D Independently from Lysosomal Degradation Inhibition

**DOI:** 10.1155/2013/846725

**Published:** 2013-07-30

**Authors:** Gessica Sala, Alessandro Arosio, Giovanni Stefanoni, Laura Melchionda, Chiara Riva, Daniele Marinig, Laura Brighina, Carlo Ferrarese

**Affiliations:** ^1^Laboratory of Neurobiology, Department of Surgery and Interdisciplinary Medicine, University of Milano-Bicocca, Via Cadore 48, 20900 Monza, Italy; ^2^PhD Program in Neuroscience, University of Milano-Bicocca, Via Cadore 48, 20900 Monza, Italy; ^3^Department of Neurology, San Gerardo Hospital, Via Pergolesi 33, 20900 Monza, Italy

## Abstract

Dysfunctions of chaperone-mediated autophagy (CMA), the main catabolic pathway for alpha-synuclein, have been linked to the pathogenesis of Parkinson's disease (PD). Since till now there is limited information on how PD-related toxins may affect CMA, in this study we explored the effect of mitochondrial complex I inhibitor rotenone on CMA substrates, alpha-synuclein and MEF2D, and effectors, lamp2A and hsc70, in a human dopaminergic neuroblastoma SH-SY5Y cell line. Rotenone induced an upregulation of alpha-synuclein and MEF2D protein levels through the stimulation of their *de novo* synthesis rather than through a reduction of their CMA-mediated degradation. Moreover, increased MEF2D transcription resulted in higher nuclear protein levels that exert a protective role against mitochondrial dysfunction and oxidative stress. These results were compared with those obtained after lysosome inhibition with ammonium chloride. As expected, this toxin induced the cytosolic accumulation of both alpha-synuclein and MEF2D proteins, as the result of the inhibition of their lysosome-mediated degradation, while, differently from rotenone, ammonium chloride decreased MEF2D nuclear levels through the downregulation of its transcription, thus reducing its protective function. These results highlight that rotenone affects alpha-synuclein and MEF2D protein levels through a mechanism independent from lysosomal degradation inhibition.

## 1. Introduction

Parkinson's disease (PD) belongs to the large category of neurodegenerative diseases caused by protein misfolding. Among the different pathogenetic mechanisms involved in the degeneration of dopaminergic neurons in PD, a central role seems to be played by the intraneuronal accumulation and aggregation of alpha-synuclein. This evidence has raised the challenge of establishing the pathogenetic role of the biological systems influencing neuronal protein homeostasis. Hence, the efficiency of cell clearance machinery has been identified as crucial for neuronal susceptibility to protein toxicity. After the demonstration that chaperone-mediated autophagy (CMA) represents the main catabolic pathway for alpha-synuclein [1, 2], it has been postulated that dysfunctions of CMA, even more than ubiquitin-proteasome system (UPS) and macroautophagy, are involved in the pathogenesis of PD. The CMA process, responsible for the selective degradation of aberrant proteins containing the consensus peptide sequence KFERQ, requires the presence of two main proteins: cytosolic and lysosomal heat shock cognate protein 70 (hsc70) and lysosomal-associated membrane protein 2A (lamp2A). Cytosolic hsc70 binds the KFERQ sequence of substrate proteins and carries them to the lysosomal membrane, where lamp2A, after interaction with cytosolic hsc70, multimerizes and forms a translocation complex with lysosomal hsc70, thus mediating the transport of the substrate protein into the lysosomal lumen. As the binding of the substrate protein to lamp2A represents the limiting step of CMA, lamp2A levels have been shown to directly correlate with CMA activity [3, 4]. Oxidative stress, accumulation of substrates, and lack of nutrients and growth factors are all conditions determining a compensatory and cytoprotective activation of CMA through an increase of lamp2A levels on lysosomal membrane [5].

The evidence of a strong correlation between the functional state of CMA and the deleterious action of alpha-synuclein has been reinforced by the demonstration that both pathogenetic mutations and overexpression of alpha-synuclein inhibit this process [1, 6]. The finding of low levels of lamp2A and hsc70 in postmortem substantia nigra of patients with sporadic PD indicates that a reduced CMA activity is likely to be a pathogenetic mechanism even in idiopathic PD [7]. Therefore, it is conceivable to assume that other CMA substrates besides alpha-synuclein contribute to neuronal death through their accumulation. In particular, recent studies identify myocyte enhancer factor 2D (MEF2D) as an important link between CMA alterations and neuronal damage associated to PD. MEF2D is a transcription factor implicated in neuronal survival, whose inactivation mediates MPTP-induced toxicity [8]; moreover, MEF2D is a specific CMA substrate that accumulates in the cytosol following CMA inhibition, with consequent reduction of its nuclear levels and thus of its transcriptional activity [9]. MEF2D is present in rodent neuronal mitochondria, where it regulates complex I activity, energy production, and oxidative cell status [10]. Postmortem studies on brain samples of patients with PD have shown a cytosolic accumulation of MEF2D [9], consistent with a condition of CMA inactivation.

Hence, the failure of CMA seems to be a pathogenetic mechanism favoring the death of dopaminergic neurons and possibly contributing to the development and progression of PD. 

While the interactions between pathogenetic forms of alpha-synuclein and CMA activity are documented, till now there is limited information on how mitochondrial dysfunctions induced by environmental toxins associated with PD may affect CMA. Results from *in vivo* studies indicate that the herbicide paraquat increases lamp2A levels and the lysosomal content of alpha-synuclein and hsc70 in mice [2], and an increase in nigral lamp2A has been demonstrated in 6-OHDA-lesioned hemiparkinsonian rats [11]. Only one *in vitro* study performed in a rat pheochromocytoma cell line demonstrated that MPP+ or acidic damage application induces lamp2A overexpression [12], and a MEF2D downregulation was found to mediate 6-OHDA-induced death in the same cell line [13]. To our knowledge an *in vitro* study assessing the effect of rotenone-induced mitochondrial complex I inhibition on CMA substrates and effectors does not exist. Therefore, this study was designed in order to clarify the effect of an acute exposure to the PD-related toxin rotenone on CMA substrates (alpha-synuclein and MEF2D) and effectors (lamp2A and hsc70) in a human dopaminergic neuroblastoma SH-SY5Y cell line.

## 2. Experimental Procedures

All reagents were obtained from Sigma-Aldrich unless otherwise stated. 

### 2.1. Cell Cultures

Human neuroblastoma SH-SY5Y cells were grown in Dulbecco's modified Eagle's medium-F12 supplemented with 10% fetal bovine serum, 100 U/mL penicillin, and 100 *μ*g/mL streptomycin, at 37°C in an atmosphere of  5% CO_2_ in air. 

### 2.2. Cytotoxicity Assays

Cytotoxicity of mitochondrial complex I inhibitor rotenone and lysosomal inhibitor ammonium chloride was assessed by MTT assay. After exposure to rotenone (from 25 to 800 nM) or ammonium chloride (from 0.05 to 40 mM) for 24 hours, SH-SY5Y cells were incubated with 0.5 mg/mL MTT in standard medium for 45 min at 37°C in an atmosphere of 5% CO_2_ in air. After cell solubilization with DMSO, cell viability was quantified (wavelength 570 nm) on a multiwell scanning spectrophotometer (Bio-Rad).

### 2.3. Whole-Cell Reactive Oxygen Species (ROS) Levels

The dye 2′,7′-dichlorofluorescein diacetate (DCF-DA) was used to quantify the levels of whole-cell ROS. After medium removal, cells were exposed to 10 *μ*M DCF-DA in Locke's buffer (154 mM NaCl, 5.6 mM KCl, 3.6 mM NaHCO_3_, 2.3 mM CaCl_2_, 5.6 mM glucose, 5 mM Hepes, 1.2 mM MgCl_2_, pH 7.4) for 45 min at 37°C in an atmosphere of 5% CO_2_ in air. Cells were washed in Locke's buffer without glucose, harvested, and lysed. Fluorescence units (FU) were quantified (excitation 488 nm, emission 525 nm) and related to the total protein content assessed using the method of Bradford.

### 2.4. Nuclei Isolation

The Nuclei EZ Prep kit was used, according to the manufacturer instructions. Briefly, after medium removal, cells were washed with ice cold PBS, harvested by scraping, and lysed in ice cold Nuclei EZ lysis buffer. Cell lysates were set on ice for 5 min and centrifuged at 500 ×g for 5 min at 4°C. The supernatant, containing cytoplasmic components, was stored at −80°C, and the nuclei pellet was resuspended in ice cold Nuclei EZ lysis buffer, set on ice for 5 min, and centrifuged at 500 ×g for 5 min at 4°C. The resulting nuclei pellet was resuspended in ice cold Nuclei EZ storage buffer, triturated, and stored at −80°C.

### 2.5. RNA Extraction and cDNA Synthesis

Total RNA was extracted using the RNeasy Mini kit (Qiagen), according to the manufacturer instructions. RNA concentration was determined spectrophotometrically at 260 nm. RNA (2000 ng) was retrotranscribed into cDNA using the SuperScript VILO cDNA Synthesis Kit (Invitrogen) at the following conditions: 10 min at 25°C and 60 min at 42°C. The reaction was terminated at 85°C for 5 min, and cDNAs were stored at −20°C. 

### 2.6. Real-Time Quantitative PCR (qPCR)

cDNAs obtained from RNA (100 ng for alpha-synuclein and lamp2A and 50 ng for hsc70) were amplified in triplicate in the ABI Prism 7500 HTSequence Detection System (Applied Biosystems) using the Platinum SYBR Green qPCR SuperMix-UDG (Invitrogen) at the following conditions: 50°C for 2 min, 95°C for 10 min, 40 cycles of 95°C for 15 sec, 60°C for 30 sec. The following primer pairs were used: alpha-synuclein-F (GCAGCCACTGGCTTTGTCAA) and alpha-synuclein-R (AGGATCCACAGGCATATCTTCCA); lamp2A-F (GCAGTGCAGATGAAGACAAC) and lamp2A-R (AGTATGATGGCGCTTGAGAC); hsc70-F (CAGGTTTATGAAGGCGAGCGTGCC) and hsc70-R (GGGTGCAGGAGGTATGCCTGTGA); beta-actin-F (TGTGGCATCCACGAAACTAC) and beta-actin-R (GGAGCAATGATCTTGATCTTCA). 

For the analysis of MEF2D mRNA levels, cDNAs obtained from 70 ng RNA were amplified in quadruplicate using TaqMan Gene Expression Assay (Applied Biosystems, assay ID: Hs00954735_m1; beta-actin assay ID: Hs99999903_m1).

For relative quantification of each target versus beta-actin mRNA, the comparative C_T_ method was used as previously described [14].

### 2.7. Western Blotting

Cell pellets were lysed, and protein concentrations were determined by Bradford's method. After denaturation, 30 *μ*g (for alpha-synuclein, MEF2D, hsc70, LC3-II, and beclin 1) or 50 *μ*g (for lamp2A) proteins were separated by electrophoresis in NuPAGE Novex 4–12% Bis-Tris gels (Invitrogen) and transferred to nitrocellulose. Blots were blocked for 1 hour, incubated overnight at 4°C with specific primary antibodies (alpha-synuclein and MEF2D, BD Biosciences, 1 : 1000 dilution; lamp2A and hsc70, Abcam, 1 : 500 and 1 : 3000 dilution, resp.; LC3B and beclin 1, Cell Signaling, 1 : 500 and 1 : 1000 dilution, resp.) and then with HRP-linked anti-mouse or -rabbit IgG for 1 hour. Beta-actin was used as internal standard for total protein extracts and cytosolic fractions and PARP for nuclear fractions. Signals were revealed by chemiluminescence, visualized on X-ray film, and quantified by GS-690 Imaging Densitometer (Bio-Rad). 

### 2.8. Statistical Analysis

All data are shown as mean ± standard deviation (SD). Statistical analysis was performed using GraphPad Prism 4.0. Two-tailed paired Student's *t*-test or one-way ANOVA, followed by Dunnett's multiple comparison test, was used to assess the significance of differences between 2 or more than 2 groups, respectively. Correlation was computed with Pearson's *r* test.

## 3. Results

### 3.1. Assessment of Rotenone Toxicity in SH-SY5Y Cells

**Figure 1:**
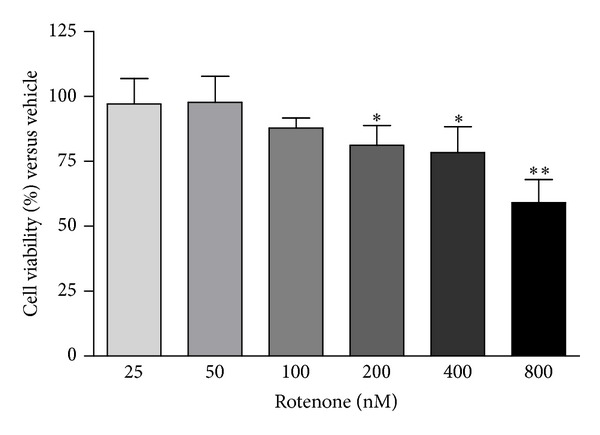
Rotenone cytotoxicity in SH-SY5Y cells. Cell viability was assessed by MTT assay after 24-hour exposure to 25–800 nM rotenone. Values are expressed as % versus vehicle. *N* = 4, repeated measures ANOVA test, followed by Dunnett's post-test; *P < 0.05; **P < 0.01 versus vehicle.

Preliminary experiments were carried out to establish the dose dependency of rotenone cytotoxicity in SH-SY5Y cells. After 24-hour exposure to rotenone concentrations ranging from 25 to 800 nM, a significant cytotoxicity was evidenced starting from 200 nM concentration (Figure 1). Based on these results, we decided to evaluate the effects of 100 and 200 nM rotenone, concentrations displaying no or mild cytotoxicity at the chosen exposure time (24 hours), on the expression of target proteins. The rotenone concentrations employed in this study are known to induce at least a 70% reduction of the mitochondrial respiration and complex I activity [15, 16]. 

Whole-cell intracellular ROS production was quantified as index of rotenone-induced oxidative stress. Rotenone exposure (200 nM for 24 hours) results in a significant 45% increase (*P* < 0.05) in whole-cell ROS levels with respect to vehicle-treated cells (0.78 ± 0.07 versus 0.54 ± 0.07 DCF FU/*μ*g prot. in rotenone versus vehicle-treated cells), as reported in the literature and in a recent published study [17].

### 3.2. Rotenone Induces Alpha-Synuclein, MEF2D, and lamp2A Transcriptional Upregulation

**Figure 2:**
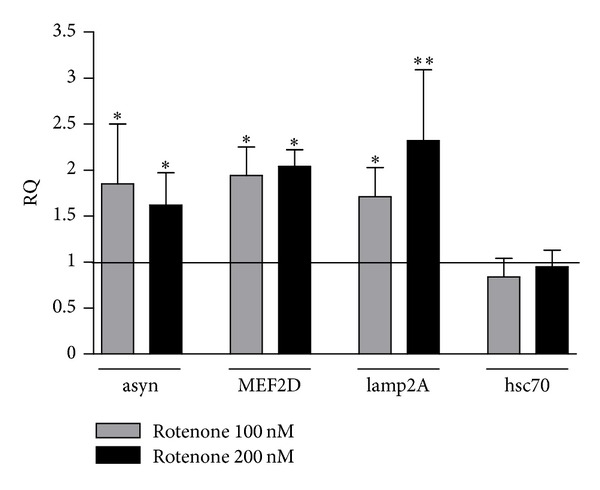
Effect of rotenone on alpha-synuclein (asyn), MEF2D, lamp2A, and hsc70 mRNA levels. Relative quantification (RQ) of asyn, MEF2D, lamp2A, and hsc70 mRNA calculated as ratio to beta-actin in SH-SY5Y treated with 100 and 200 nM rotenone for 24 hours, expressed as fold change versus vehicle (RQ = 1). *N* = 4, repeated measures ANOVA test, followed by Dunnett's post-test; *P < 0.05; **P < 0.01 versus vehicle.

Following 24-hour treatment with 100 and 200 nM rotenone, mRNA levels codifying for alpha-synuclein, MEF2D, lamp2A, and hsc70 were quantified in SH-SY5Y cells by real-time PCR.  Figure 2 summarizes the relative quantification (RQ) of each target normalized to beta-actin in cells exposed to the toxic as compared to vehicle-treated cells.

Rotenone induced a significant 1.85- and 1.62-fold increase (*P* < 0.05) of mRNA codifying for alpha-synuclein and a significant 1.94- and 2.04-fold increase (*P* < 0.05) of MEF2D mRNA (Figure 2). The same rotenone concentrations were also able to produce a significant 1.71- (*P* < 0.05) and 2.32-fold increase (*P* < 0.01) of lamp2A mRNA levels at the concentration of 100 and 200 nM, respectively, while no change was found in hsc70 gene expression. 

### 3.3. Rotenone Induces Alpha-Synuclein and MEF2D, but Not lamp2A and hsc70, Protein Expression

**Figure 3:**
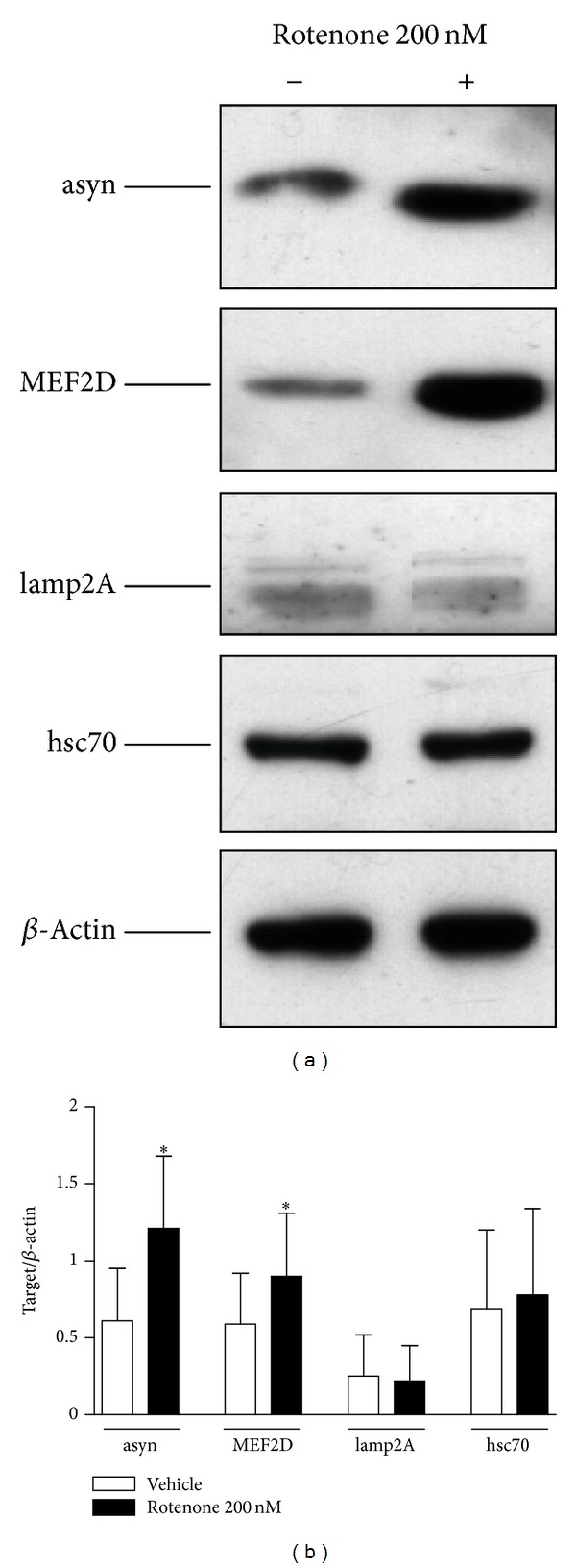
Effect of rotenone on alpha-synuclein (asyn), MEF2D, lamp2A, and hsc70 protein expression. (a) Representative Western blot analysis of asyn, MEF2D, lamp2A, and hsc70 in SH-SY5Y cells in basal conditions and after exposure to 200 nM rotenone for 24 hours. The immunoreactivity of beta-actin, used as internal standard, was also shown. (b) Mean values of asyn, MEF2D, lamp2A, and hsc70 protein expression, expressed as ratio between each target and beta-actin optical density. *N* = 4, paired Student's *t*-test; *P < 0.05 versus vehicle.

Western blot analyses showed that 24-hour treatment with 200 nM rotenone causes a marked and significant increase of alpha-synuclein (+98%, *P* < 0.05) and MEF2D (+52%, *P* < 0.05) proteins as compared to vehicle-treated cells (Figures 3(a) and 3(b)), while it does not affect lamp2A and hsc70 protein expression.

### 3.4. Rotenone Induces an Increase of MEF2D Protein Expression in the Nucleus

**Figure 4:**
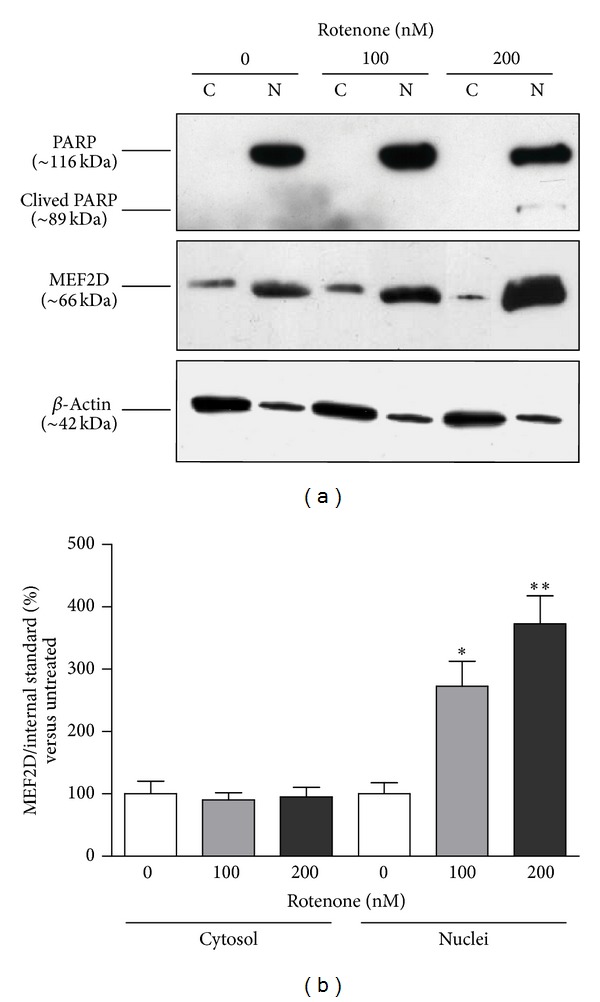
Effect of rotenone on intracellular localization of MEF2D. (a) Representative Western blot analysis of MEF2D in the cytosolic (C) and nuclear (N) fractions obtained from SH-SY5Y cells in basal conditions and after exposure to 100 and 200 nM rotenone for 24 hours. The immunoreactivities of beta-actin, used as internal standard for the cytosol, and PARP, used as internal standard for the nuclei, were also shown. (b) Mean values of MEF2D protein expression in cytosolic and nuclear fractions, expressed as ratio to beta-actin and PARP, respectively. *N* = 4, repeated measures ANOVA test, followed by Dunnett's post-test; *P < 0.05; **P < 0.01 versus vehicle.

The observed increase of  MEF2D protein expression following rotenone exposure prompted us to clarify the intracellular localization of this increase, based on the evidence that MEF2D physiologically translocates from the nucleus to the cytosol for its degradation. Therefore, after treatment with 100 and 200 nM rotenone, nuclear and cytosolic cell fractions were isolated for MEF2D protein assessment. Under basal conditions, MEF2D immunoreactivity was almost exclusively detected in the nuclear fraction, with only a faint immunoreactive signal in the cytosol, in line with the physiological localization of this nuclear transcription factor. Cytosolic and nuclear MEF2D immunoreactivity was normalized for beta-actin and PARP expression, respectively. We observed a marked increase of  MEF2D protein levels after 100 nM (+172%, *P* < 0.05) and 200 nM (+272%, *P* < 0.01) rotenone treatment in the nuclear fraction, with no change in the cytosolic fraction (Figures 4(a) and 4(b)). As shown in Figure 4(a), an immunoreactive signal corresponding to the cleaved form of PARP appears in cell exposed to 200 nM rotenone, indicating the presence of an apoptotic induction.

### 3.5. Rotenone Induces Autophagosome Accumulation

**Figure 5:**
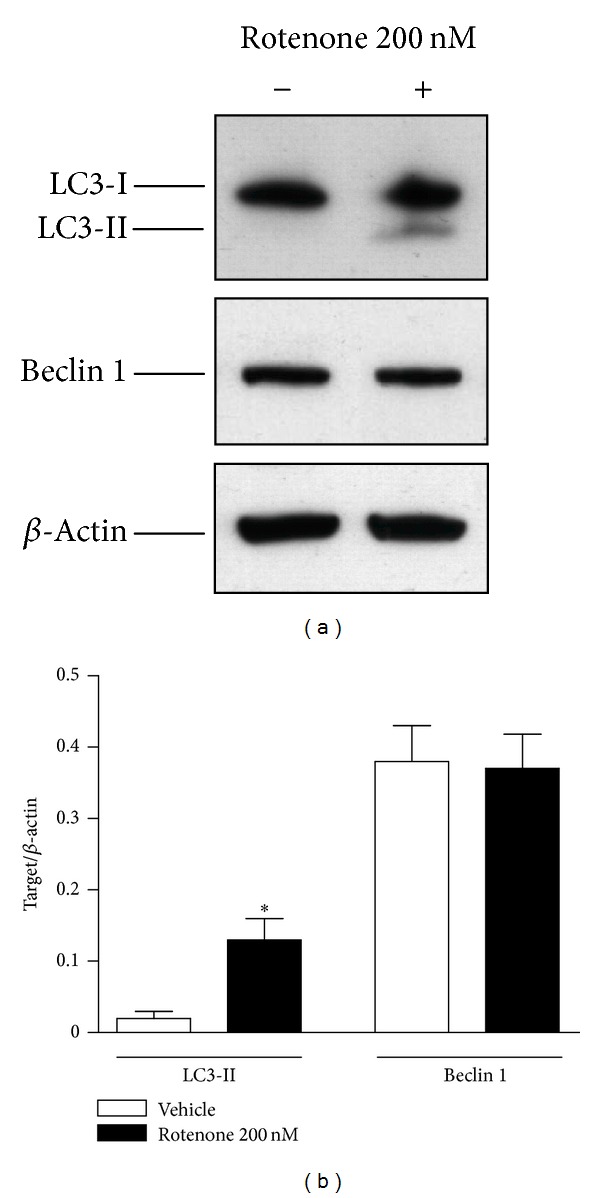
Effect of rotenone on LC3-II and beclin 1 protein expression. (a) Representative Western blot analysis of LC3-II and beclin 1 in SH-SY5Y cells in basal conditions and after exposure to 200 nM rotenone for 24 hours. The immunoreactivity of beta-actin, used as internal standard, was also shown. (b) Mean values of LC3-II and beclin 1 protein expression, expressed as ratio between each target and beta-actin optical density. *N* = 3, paired Student's *t*-test; *P < 0.05 versus vehicle.

To evaluate the effect of rotenone on macroautophagy, the expression of specific macroautophagy proteins was evaluated by Western blot.  Figure 5 shows that 24-hour treatment with 200 nM rotenone causes a significant increase of LC3-II, with no change in beclin 1.

### 3.6. Effect of Lysosome Inhibition on Alpha-Synuclein, MEF2D, lamp2A, and hsc70 Expression

**Figure 6:**
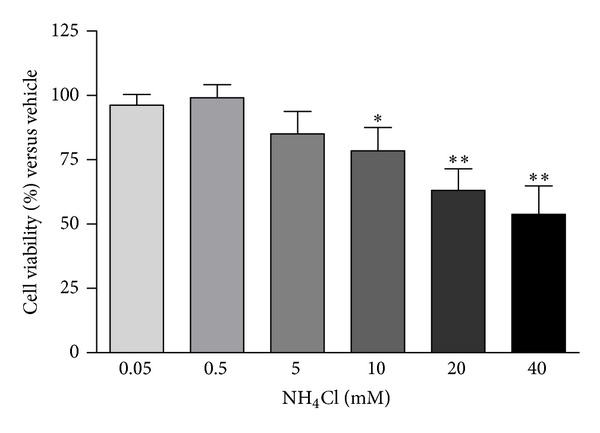
Ammonium chloride (NH_4_Cl) cytotoxicity in SH-SY5Y cells. Cell viability was assessed by MTT assay after 24-hour exposure to 0.05–40 mM NH_4_Cl. Values are expressed as % versus vehicle. *N* = 4, repeated measures ANOVA test, followed by Dunnett's post-test; *P < 0.05; **P < 0.01 versus vehicle.

Cell viability assays performed after 24-hour exposure to ammonium chloride concentrations ranging from 0.05 to 40 mM evidenced a significant cytotoxicity starting from 10 mM concentration (Figure 6). Based on these results, we evaluated the effects of 5 and 10 mM ammonium chloride on the expression of target proteins.

**Figure 7:**
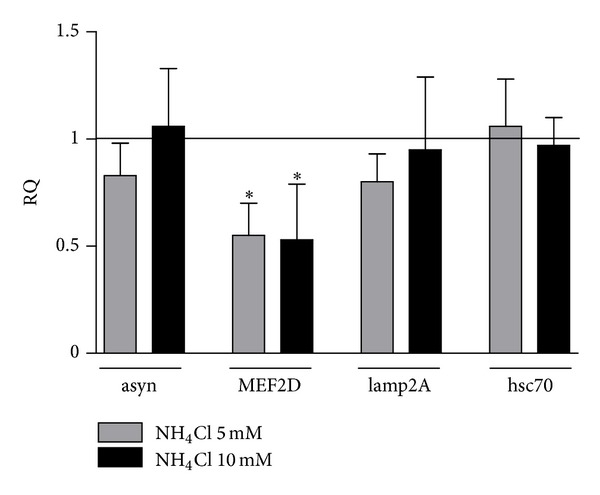
Effect of ammonium chloride (NH_4_Cl) on alpha-synuclein (asyn), MEF2D, lamp2A, and hsc70 mRNA levels. Relative quantification (RQ) of asyn, MEF2D, lamp2A, and hsc70 mRNA calculated as ratio to beta-actin in SH-SY5Y treated with 5 and 10 mM NH_4_Cl for 24 hours, expressed as fold change versus vehicle (RQ = 1). *N* = 4, repeated measures ANOVA test, followed by Dunnett's post-test; *P < 0.05; **P < 0.01 versus vehicle.

Differently from rotenone, 24-hour cell treatment with 5 and 10 mM ammonium chloride did not produce any significant transcriptional modification of alpha-synuclein, while resulted in a decrease of about 0.5-fold (*P* < 0.05) of MEF2D mRNA levels (Figure 7). No effect was evidenced on lamp2A and hsc70 mRNA levels (Figure 7).

**Figure 8:**
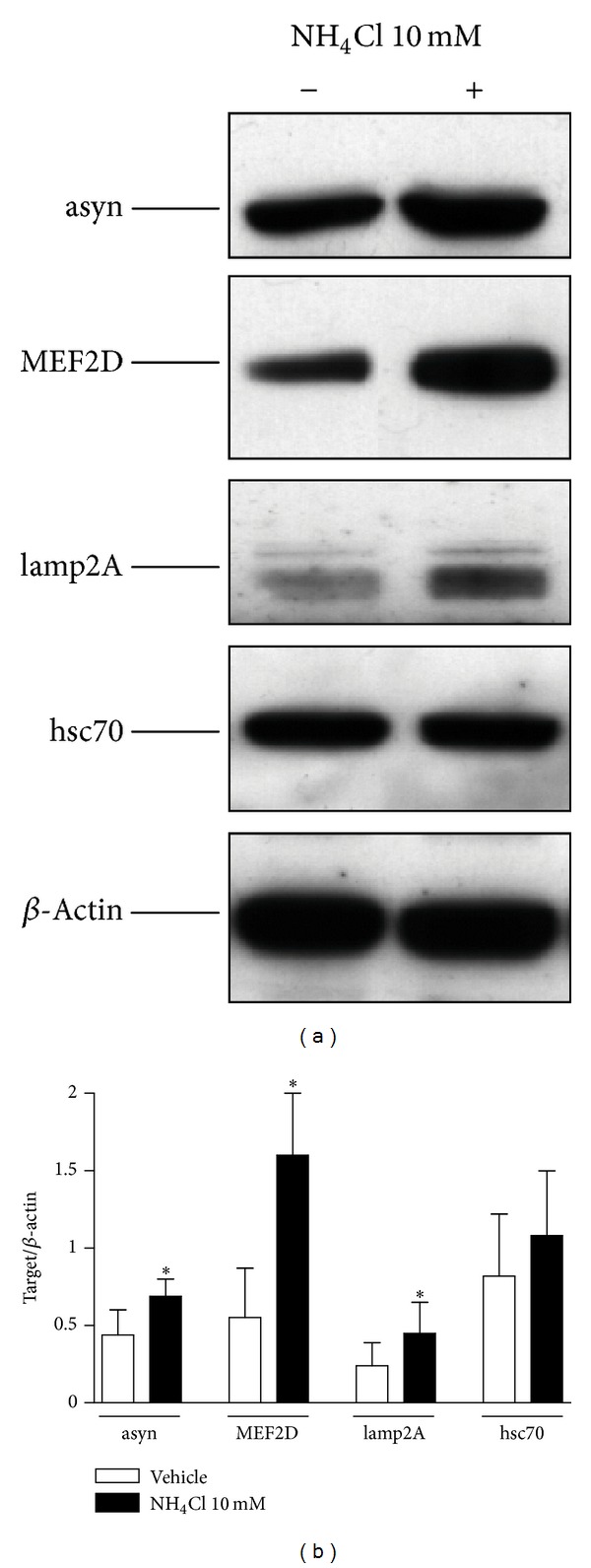
Effect of ammonium chloride (NH_4_Cl) on alpha-synuclein (asyn), MEF2D, lamp2A, and hsc70 protein expression. (a) Representative Western blot analysis of asyn, MEF2D, lamp2A, and hsc70 in SH-SY5Y cells in basal conditions and after exposure to 10 mM NH_4_Cl for 24 hours. The immunoreactivity of beta-actin, used as internal standard, was also shown. (b) Mean values of asyn, MEF2D, lamp2A, and hsc70 protein expression, expressed as ratio between each target and beta-actin optical density. *N* = 4, paired Student's *t*-test; *P < 0.05 versus vehicle.

Western blot analyses showed that 24-hour treatment with 10 mM ammonium chloride significantly increases alpha-synuclein (+57%, *P* < 0.05), MEF2D (+190%, *P* < 0.05), and lamp2A (+87%, *P* < 0.05) protein expression, with no effect on hsc70 (Figures 8(a) and 8(b)).

**Figure 9:**
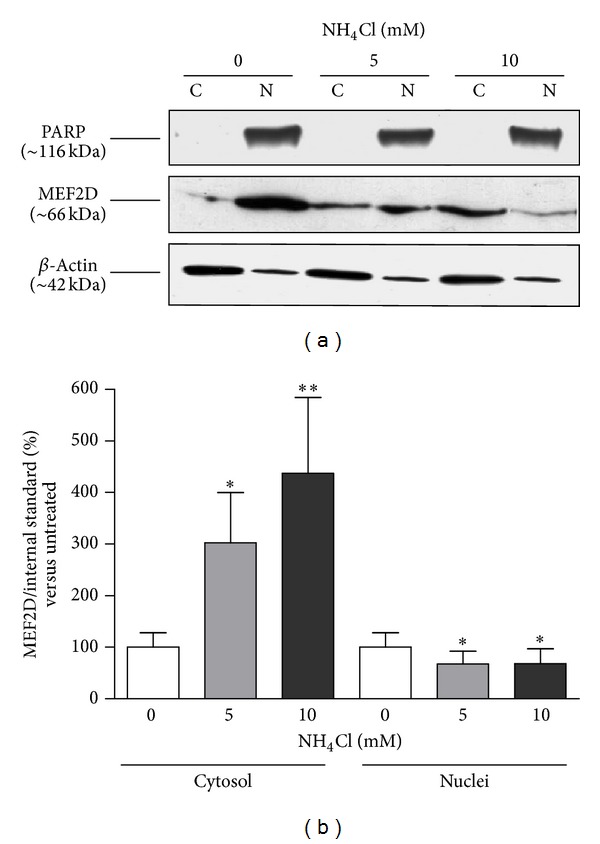
Effect of ammonium chloride (NH_4_Cl) on intracellular localization of MEF2D. (a) Representative Western blot analysis of MEF2D in the cytosolic (C) and nuclear (N) fractions obtained from SH-SY5Y cells in basal conditions and after exposure to 5 and 10 mM NH_4_Cl for 24 hours. The immunoreactivities of beta-actin, used as internal standard for the cytosol, and PARP, used as internal standard for the nuclei, were also shown. (b) Mean values of MEF2D protein expression in cytosolic and nuclear fractions, expressed as ratio to beta-actin and PARP, respectively. *N* = 4, repeated measures ANOVA test, followed by Dunnett's post-test; *P < 0.05; **P < 0.01 versus vehicle.

The assessment of MEF2D expression in both nuclear and cytosolic fractions indicates a marked increase in the cytosolic MEF2D expression after exposure to 5 mM (+200%, *P* < 0.05) and 10 mM (+330%, *P* < 0.01) ammonium chloride, paralleled by a significant reduction (−30%, *P* < 0.05) of its nuclear levels (Figures 9(a) and 9(b)).

Table 1 summarizes results obtained in this study.

## 4. Discussion

In this *in vitro* study performed in a human neuroblastoma SH-SY5Y cell line we assessed the effects of an acute exposure to rotenone, an inhibitor of the mitochondrial complex I activity able to reproduce the mitochondrial dysfunction typical of PD, on the expression of 2 critical protein associated to PD, alpha-synuclein, and MEF2D. We also investigated whether the observed effects of rotenone on alpha-synuclein and MEF2D proteins were due to an increased protein synthesis rather than to an impaired degradation. To reach this aim, alpha-synuclein and MEF2D mRNA levels were quantified, as well as the expression of the 2 key-proteins of CMA, the main catabolic pathway of alpha-synuclein and MEF2D.

To further clarify the specific mechanisms underlying the effects of rotenone on alpha-synuclein and MEF2D, all the parameters investigated after rotenone exposure were also assessed in an experimental paradigm characterized by the inhibition of the lysosomal protease activity. The lysosome inhibition was obtained by exposing cells to ammonium chloride, a compound able to inhibit the activity of lysosomal proteases, thus mimicking a block of all catabolic pathways that end with the lysosome-mediated substrate degradation, including macroautophagy and CMA.

Results obtained in this study demonstrate that rotenone does not cause alterations of the key-regulators of CMA, lamp2A, and hsc70, although it induces a *de novo* synthesis of the lysosomal CMA receptor lamp2A, as indicated by the increase of lamp2A mRNA levels. While the increased lamp2A mRNA levels observed after rotenone exposure agree with the previous findings obtained from *in vivo* studies using paraquat or 6-OHDA [2, 11] and are in line with the knowledge that CMA activation induced by oxidative stress is caused by an increased lamp2A synthesis [5], the unchanged lamp2A protein levels are unexpected. Certainly, we cannot exclude the possibility that Western blot analyses performed using the unique antibody currently commercially available (Abcam 18528) able to react against lamp2A in human samples are not sufficiently sensitive to highlight putative mild difference of lamp2A protein expression. Supporting this hypothesis, it has been recently reported that the detection of lamp2A immunoreactive signal is difficult and characterized by the presence of high nonspecific signals associated with the use of the same antibody used in this study [18].

To verify the specificity of our lamp2A signals, we performed blocking experiments using an immunizing peptide (Abcam 23322, 1 *μ*g/mL), and we found that lamp2A staining completely disappears, thus confirming the specificity of the immunoreactive signal. Furthermore, to overcome this methodological limitation, in this study, we also performed a quantitative analysis of this and other targets by real-time PCR, which may be a more sensitive and specific method. Alternatively, the unchanged lamp2A protein levels observed after rotenone exposure might suggest the existence of an increased lamp2A degradation induced by rotenone and never described so far.

Our results also indicate that rotenone is able to increase mRNA and protein levels of CMA substrates, alpha-synuclein, and MEF2D. While chronic exposure to rotenone was already demonstrated to induce alpha-synuclein accumulation both in animal and *in vitro* models [19, 20], till now no data on alpha-synuclein *de novo* synthesis were reported. Using the same experimental conditions, Kalivendi et al. [21] showed that rotenone induces alternative splicing of alpha-synuclein mRNA, with a dose-dependent increase in the expression of a shorter protein isoform. On the other hand, a very recent report [22] proposes that alpha-synuclein may confer resistance to low-dose rotenone toxicity, while an increased resistance to rotenone toxicity has been observed in lymphoblastoid cell lines derived from PD patients with SNCA duplication [23]. In this study, we also verified the involvement of macroautophagy in mediating rotenone-induced alpha-synuclein increase. To this purpose, mRNA and protein alpha-synuclein levels were evaluated in the presence of the macroautophagy inhibitor 3-methyladenine (3-MA, 5 mM, 1 hour before rotenone treatment). 3-MA alone induces an increase of alpha-synuclein protein levels and a compensatory reduction of its transcription (data not shown). Furthermore, rotenone, even in presence of 3-MA, results in an increase of alpha-synuclein mRNA levels and protein levels (data not shown), suggesting that the observed results are independent from macroautophagy inhibition.

Moreover, our results represent the first evidence for a role of rotenone on MEF2D regulation. This preliminary observation needs to be further explored through functional studies aimed to clarify the precise and unknown mechanistic link between rotenone and MEF2D. Collectively, these findings support the hypothesis that rotenone, at the incubation time and concentrations used in this study, induces an increased expression of both alpha-synuclein and MEF2D not directly imputable to CMA alterations. Furthermore, according to previous studies demonstrating a macroautophagy induction after rotenone exposure [24–26], in our experimental conditions we observed an LC3-II increase, consistent with an autophagosome accumulation induced by rotenone, with no appreciable change in beclin 1 protein levels.

Another interesting novelty of this study is derived from the analysis of the intracellular localization of the transcription factor MEF2D. In fact, we found higher MEF2D protein expression in the nucleus after rotenone exposure, with no change in the cytosolic MEF2D levels. These results, together with the observed upregulation of MEF2D mRNA levels, suggest that MEF2D increase is not due to a defect in its catabolic pathway but reflects a *de novo* synthesis. This study highlights for the first time a role for rotenone in inducing an upregulation of the neuronal transcription factor MEF2D that can be interpreted as a compensatory and prosurvival cell attempt possibly contrasting rotenone toxicity. The demonstration of the existence of a time window that follows the toxic insult in which cells seem to counteract the damage by inducing the transcription of protective factors can be potentially useful to intervene with a neuroprotective therapy in PD patients. Furthermore, the identification of such a role for MEF2D indicates that its upregulation might represent a new promising therapeutic strategy for PD, as recently demonstrated in an *in vitro* PD model [27].

The comparison of results obtained after exposure to rotenone with respect to ammonium chloride highlights that, although both toxics cause an increase of the protein levels of CMA substrates, the mechanisms responsible for these increases are different. As a matter of fact, differently from rotenone, ammonium chloride causes an increase of MEF2D levels in the cytosolic fraction, with a concomitant reduction of its nuclear levels, consistent with an inhibition of the lysosome-mediated protein degradation and in line with previous findings [9]. The analysis of MEF2D mRNA levels performed for the first time in this study definitively demonstrates that ammonium chloride leads to a transcriptional downregulation of MEF2D, likely representing an adaptative response to the cytosolic protein accumulation. The observed increase of alpha-synuclein protein levels after ammonium chloride exposure, ascribable to an inhibition of the lysosomal degradation induced by the toxic, agrees with findings of a recent study showing that ammonium chloride leads to an increase of alpha-synuclein levels and release in exosomes and a concomitant increase of alpha-synuclein transmission to recipient cells [28]. The increase of lamp2A protein levels evidenced after ammonium chloride exposure may be interpreted as a compensatory reduced degradation of lamp2A in response to the presence of CMA substrate accumulation, as already reported [3]. Moreover, our results indicate that ammonium chloride does not affect hsc70 expression, consistent with the knowledge that this CMA carrier is abundant in the cytosol; hence it does not represent, differently from lamp2A, a rate-limiting protein of CMA pathway, except for its lysosomal level [5].

## 5. Conclusions

Ultimately, this study demonstrates that rotenone induces an upregulation of alpha-synuclein and MEF2D protein levels through the stimulation of their *de novo* synthesis (as indicated by the increased mRNA levels) rather than through a reduction of their CMA-mediated degradation (as indicated by unchanged levels of the main CMA machinery effectors, lamp2A and hsc70). In particular, we demonstrated that increased MEF2D transcription results in higher nuclear protein levels, which are known to promote protective mechanisms against mitochondrial dysfunction and oxidative stress conditions.

Conversely, ammonium chloride induces the cytosolic accumulation of both alpha-synuclein and MEF2D, as the result of the inhibition of their lysosome-mediated degradation, while the toxin reduces specifically MEF2D nuclear levels (through the downregulation of its transcription), thus leading to a reduction of its protective function.

Taken together our results highlight that PD-related toxins affect alpha-synuclein and MEF2D protein levels through distinct mechanisms and in particular support the use of MEF2D expression and intracellular localization as a reliable “sensor” of the perturbation of cellular homeostasis in PD *ex vivo* and *in vitro* models.

## Figures and Tables

**Table 1:** Summary of results.

	Alpha-synuclein		MEF2D			lamp2A		hsc70	
	mRNA	Protein	mRNA	Protein		mRNA	Protein	mRNA	Protein
Rotenone	↑	↑	↑	↑		↑	=	=	=
				N	C				
				↑	=				

NH_4_Cl	=	↑	↓	↑		=	↑	=	=
				N	C				
				↓	↑				

N: nuclei, C: cytosol.

## Abbreviations

CMA: Chaperone-mediated autophagy
PD: Parkinson's disease
UPS: Ubiquitin-proteasome system
MEF2D: Myocyte enhancer factor 2D
hsc70: Heat shock cognate protein 70
lamp2A: Lysosomal-associated membrane protein 2A.
